# INSIGHTS FROM A NATIONAL COMPARISON STUDY ON MENTAL HEALTH CONDITIONS OF AUSTRALIAN AGED CARE USERS

**DOI:** 10.1093/geroni/igae098.4224

**Published:** 2024-12-31

**Authors:** Lok Man Leung, Elsie Yan

**Affiliations:** The Hong Kong Polytechnic University, Hong Kong, Hong Kong; The Hong Kong Polytechnic University, Hong Kong, Hong Kong

## Abstract

**Background:**

The mental health status of aged care service users has important implications for service planning and care outcomes. Objective: This study examines the prevalence of mental health conditions among Aboriginal and Torres Strait Islanders aged 50 and above who accessed aged care services in Australia between 2017 and 2022. Comparison was made between Home Care(HC) users and Residential Aged Care(RAC). Method: Data were obtained from the Australian Institute of Health and Welfare(AIHW) database on Mental Health in Aged Care(n=10,535 for HC and n = 6,757 for RAC users). Mental health conditions were assessed by Aged Care Eligibility Assessments and, where applicable, the Geriatric Depression Scale(GDS), Kessler-10(K10), and Cornell Scale for Depression in Dementia(CSDD).

**Results:**

Mental health conditions were more prevalent in RAC than HC users (58.6% vs. 31.1%, X²=1276.41, p<.01), with mood disorders being the most common, affecting 43.1% of RAC and 20.9% of HC users (X²=970.89,p <.01). The same pattern was observed for anxiety/stress disorders (26.5% vs. 11.2%, X² = 682.97, p<.01) and psychotic disorders(6.2% vs. 4.1%, X² = 34.99, p<.01). Over the five years (2017–2022), a steady increase in recorded mental health conditions was observed in both settings (from 21.0% to 34.5% for HC and 56.0% to 58.4% for RAC). The increase is particularly sharp for mood disorders (from 12.3% to 24.1% for HC and 40.2% to 42.7% for RAC).

**Conclusion:**

These findings highlight the need for targeted mental health interventions in residential care. Further research should develop interventions tailored to the specific needs of this group.

